# Correlation of peripheral CD4+GranzB+CTLs with disease severity in patients with primary Sjögren’s syndrome

**DOI:** 10.1186/s13075-021-02632-6

**Published:** 2021-10-12

**Authors:** Qi Wang, Nan Che, Chengyin Lu, Xiaoxuan Sun, Yanyan Wang, Qiang Wang, Wenfeng Tan, Lanlan Zhou, Xiaojun Zhang, Dong Xu, Lei Gu, Miaojia Zhang

**Affiliations:** 1 Department of Rheumatology, The First Affiliated Hospital of Nanjing Medical University, Nanjing, China; 2 Department of Rheumatology, Peking Union Medical College Hospital, Beijing, China

**Keywords:** Primary Sjögren’s syndrome, CD4+ T cells, CD4+GranzB+cytotoxic T cells, ESSDAI, Extraglandular manifestations

## Abstract

**Introduction:**

Primary Sjögren’s syndrome (pSS) is a chronic systemic autoimmune disease which has focal lymphocytic infiltration including a majority of CD4+ T cells. This study was to investigate the correlation of peripheral granzyme B (GranzB)-expressing CD4+ T cells with disease severity and histological lesion in patients with pSS.

**Methods:**

We recruited 116 pSS and 46 health control (HC) using flow cytometry to examine the percentage of CD4+GranzB+CTLs in the peripheral blood, and immunofluorescence to test their expression in the labial gland.

**Results:**

The percentage of CD4+GranzB+CTLs was significantly upregulated in pSS than in HC (7.1 ± 4.9% vs 3.1 ± 1.9%, *p* < 0.0001) and positive correlation with ESSDAI. The frequency of them was markedly higher in pSS with extraglandular manifestations. After excluding the other risk factors associated with pSS, they were still related to ESSDIA and extraglandular manifestations independently (*p* < 0.05), and they are the risk factor of extraglandular involvement (odds ratio = 1.928). Moreover, they could be observed in the LSGs. ROC curve analysis indicated that the area under the curve (AUC) of CD4+GranzB+CTLs was 0.796 to predict the activity of pSS and 0.851 to presume extraglandular manifestations. The best diagnostic cutoff point was 4.865 for pSS patients.

**Conclusion:**

In this study, we provide new evidence indicating the involvement of CD4+GranzB+CTLs over activation in the pathophysiology of pSS, which may serve as a new biomarker to evaluate the activity and severity of pSS.

**Supplementary Information:**

The online version contains supplementary material available at 10.1186/s13075-021-02632-6.

## Introduction

Primary Sjögren’s syndrome (pSS) is a chronic systemic autoimmune disease, which is characterized by a lympho-plasmocytic infiltration and a progressive destruction of lachrymal and salivary glands, resulting in ocular and mouth dryness. At least a third of pSS patients developed extraglandular manifestations, such as cutaneous, pulmonary, neurological, or renal damage, which could severely affect the quality of life and might cause fatal consequences [1, 2]. The pathogenesis of this disease is still unknown. It has been shown that both innate and adaptive immune systems are participated in causation of pSS. They are possibly triggered by hormonal factors and viral infections in a genetically susceptible host [3]. A variety of immune cell populations are involved in the pathogenesis of pSS, such as dendritic cells, macrophages, T cells, and B cells [4–6].

The typical histological feature of the minor salivary glands (MSG) in pSS is the presence of lymphocytes which infiltrate around the ductal epithelium [7]. The ratio of dendritic cells, macrophages, T cells, and B cells varies according to the severity of the lesion. Large histopathological evidences found most of T cells infiltrated in the MSG of pSS patients are CD4+ T cells, distributed at the periphery of the lesion, and their number is decreasing with infiltrate severity ranging from 57% of total T cells in advanced lesions to 70% in mild lesion, whereas B lymphocytes are increased and located centrally or within ectopic germinal center (GC) [8]. Thus, it follows that CD4+ T lymphocytes may be the initial cells at the early stage of immune response in the pathogenesis of pSS.

CD4+ T cells with cytotoxic activity (CD4+CTLs) are a specialized CD4+ T cell subset which is distinguished by their ability to secrete perforin and granzyme B (GranzB) to kill the target cells in an MHC class II-restricted fashion [9]. These cells were known about as direct effectors in anti-tumor immunity and viral infections through their perforin/granzyme-mediated cytolytic activity and then contribute to cytolysis of tumors or virally infected targets [10].

As early as the 1990s, some studies have found that epithelial cells damage in MSG biopsies of pSS patients is mediated by cytotoxic T lymphocytes (CTLs) through the secretion of perforin and granzyme, then resulting in subsequent destruction of epithelial cells [11]. Moreover, several studies have shown that the epithelial cells in pSS present various different phenomena, such as MHC class II over-expression [12]. The existence of CD4+ class II-restricted CTLs was investigated using in situ double immunohistochemistry in MSG from pSS and expressed perforin mRNA [13].

Recently, Maehara et al. [14] showed that the pathogenesis of IgG4-related dacryoadenitis and sialoadenitis (IgG4-DS) was associated with CD4+GZMA+CTLs which infiltrated in tissue and secreted IFN-γ. As IgG4-DS was once categorized as a subtype of pSS, we suppose there might be some histopathological similarities between these two diseases. However, whether CD4+CTLs are involved in the development of the pSS and the pathomechanism of remains unclear.

The purpose of our study was to explore the possible involvement of CD4+CTLs in the development of pSS. Therefore, the epidemiological features, symptoms, organ involvement, and the correlation of CD4+CTLs with disease activity and clinical manifestations in pSS patients have been frequently described.

## Methods

### Patients and controls

Blood samples were obtained from pSS patients and healthy controls (HCs) admitted to the Department of Rheumatology, the First Affiliated Hospital of Nanjing Medical University (i.e., Jiangsu Province Hospital), China, between September 2017 and June 2019. HCs were people who were admitted to the hospital for dry mouth but could not diagnose any disease. All pSS patients fulfilled the American-European Consensus Group for pSS diagnosis 2002/2016 criteria [15, 16]; individuals with infections, malignant tumors, or other rheumatic diseases were excluded from the study. All patients could not use glucocorticoid ≥ 20 mg/day in the past 1 month and did not use any immunosuppressant in the past 3 months. Clinical and laboratory information obtained at the time of serum sampling included age, gender, antinuclear antibodies (ANA), anti-Ro/SSA antibody (A-SSA), anti-La/SSB antibody (A-SSB), immunoglobulin G (IgG), immunoglobulin M (IgM), immunoglobulin A (IgA), complement 3 (C3) and C4, erythrocyte sedimentation rate (ESR), rheumatoid factor (RF), and the percentage of lymphocyte subpopulation. The European League Against Rheumatism (EULAR) SS Disease Activity Index (ESSDAI) score and SS Patient Reported Index (ESSPRI) score was assessed [17]. In the meantime, we detected the frequency of CD4+GranzB+CTLs in pSS patients. In addition, the numbers of cellular infiltration per 4 mm^2^ of tissue (focus index, FI) were measured in some pSS patients and HCs. This research was in compliance with the Declaration of Helsinki. The study protocols and the consent forms were approved by the Research Ethics Committee of Jiangsu Province Hospital (approval number 2017-SR-121) and written informed consent was obtained from each individual. Detailed clinical characteristics could be seen in Table 1.

**Table 1:** Characteristics of pSS patients and the control subjects

	pSS (*n* = 116)	Control (*n* = 46)
Age (years)	47.8 ± 13.8	46.5 ± 12.1
Sex (female/male)	110/6	42/4
Disease duration (months)	49.6 ± 5.4	–
Interstitial lung disease (ILD), *n* (%)	17 (14.6)	–
Fever, *n* (%)	1 (0.9)	–
Hypocytosis, *n* (%)	29 (25.0)	–
Arthritis, *n* (%)	5 (4.3)	–
Glandular swelling, *n* (%)	3 (2.5)	–
Purpura, *n* (%)	5 (4.3)	–
Anemia, *n* (%)	7 (6.0)	–
Leukopenia, *n* (%)	15 (12.9)	–
Thrombocytopenia, *n* (%)	10 (8.6)	–
Lymphopenia, *n* (%)	7 (6.0)	–
Renal disease, *n* (%)	9 (7.7)	–
Autoimmune liver dysfunction, *n* (%)	3 (2.5)	–
Pulmonary arterial hypertension (PAH), *n* (%)	3 (2.5)	–
ANA positive	112 (96.6)	–
A-SSA positive	98 (84.5)	–
A-SSB positive	44 (37.9)	–
IgG (g/L)	18.8 ± 6.0	–
IgA (g/L)	3.3 ± 1.4	–
IgM (g/L)	1.6 ± 0.9	–
C3 (g/L)	1.0 ± 0.2	–
C4 (g/L)	0.2 ± 0.1	–
ESR (mm/H)	30.1 ± 26.2	–
RF positive	46 (39.7)	–
CD4+ T cell%	36.2 ± 9.6	
CD8+ T cell%	27.4 ± 10.1	
NK cell%	12.4 ± 7.0	
CD19 cell%	14.4 ± 8.3	
ESSDIA	0 to 24 (4.7 ± 4.8)	–
ESSPRI	0 to 7.3 (3.7 ± 1.7)	
Focus Index (1–3)	0 to 7 (2.0 ± 1.5)	–

*pSS* primary Sjogren’s syndrome, *ESSDAI* EULAR Sjogren’s syndrome Disease Activity Index, *ESSPRI* EULAR Sjögren’s Syndrome Patient Reported Index, *C3* complement 3, *C4* complement 4, *CRP* C-reactive protein, *ANA* antinuclear antibody, *ESR* erythrocyte sedimentation rate, *Focus Index* the number of foci per 4 mm^2^ of tissue. Values are expressed as mean ± standard deviation

### Flow cytometry for CD4+GranzB+CTLs and CD8+GranzB+CTLs

Blood samples were collected in heparin anticoagulant tube, transferred them to the centrifuge tubes, added Ficoll into the tubes, isolated, and collected the peripheral blood mononuclear cell (PBMC). The cell suspension was labeled with the following monoclonal antibodies: anti-human CD4-FITC (Biolegend) and CD8-PE-Vio770 (Miltenyi). For surface marker staining like CD4 and CD8, cells were maintained in the dark at 4 °C for 30 min. While for intracellular staining of granzyme B, cells were fixed and permeabilized with the Foxp3-staining kit (eBioscience) according to the manufacturer’s guidelines. After permeabilization, cell suspension was incubated in the dark at 4 °C for 20 min with anti-Granzyme B-PE (Miltenyi).

### Labial salivary gland biopsy

One hundred and three cases in the 116 cases (88.8%) of pSS patients had labial salivary gland (LSG) biopsy specimens, and in which, 91 cases matched the histological criteria for a diagnosis of pSS [18] and had obvious lymphocytic infiltration (focus index ≥ 1). The biopsies were performed for routine diagnostic purposes after acquiring the patient’s consent.

### Histologic and immunofluorescence analyses for CD4+GranzB+CTLs

There were also 42 cases of pSS patients’ LSG specimens having the immunohisto-chemical staining for CD4+GranzB+CTLs. Three-micrometer sections of the labial gland frozen in OCT were fixed in acetone, rehydrated in PBS, and blocked with 10% normal goat serum. To identify cytotoxic cells, the double staining was performed on the following combinations of primary antibodies: CD4 (ab133616, Abcam, at a dilution of 1:100)/granzyme B (MCA2120, BIO-RAD, at a dilution of 1:50). All secondary antibodies were goat. CD4 was detected using anti-Rabbit IgG H&L conjugated to Alexa Fluor® 488 (FMS), and granzyme B with anti-Mouse IgG H&L Alexa Fluor® 594 (Abcam). For negative controls, primary antibodies were omitted. Images of immunofluorescence staining were captured and visualized using a Zeiss LSM 510 confocal microscopy equipped with Cell Profiler imaging software; identical exposure times were used for each individual marker in the pSS patients and healthy control samples.

### Statistical analysis

IBM SPSS 22.0 was used for data analysis. Before the analysis, hypothesis testing was performed using normal probability plots to confirm the normal distribution of quantitative data (all *p* > 0.05), which were recorded as mean ± standard deviation, while qualitative data were recorded as ratios. The two sets of measurement data were compared by Student’s *t*-test, while comparison of categorical data was done by Pearson’s chi-square test. Age, gender, and factors related to pSS were included in the multivariate and logistic regression analysis. The backward stepwise entry method (LR method) was used to screen relevant factors affecting response to CD4+GranzB+CTLs and CD8+GranzB+CTLs. The receiver operating characteristic (ROC) curve was used to evaluate the diagnostic value of CD4+GranzB+CTLs in predicting response to the disease activity and severity of pSS. The maximum value of the Youden index was used as the optimal diagnostic threshold to calculate sensitivity and specificity. A two-tailed *p* value of 0.05 was considered significant.

## Results

### The percentage of CD4+GranzB+CTLs was significantly upregulated in pSS patients than in healthy controls and positive correlation with ESSDAI in pSS patients but had no correlation with ESSPRI

**Fig. 1:**
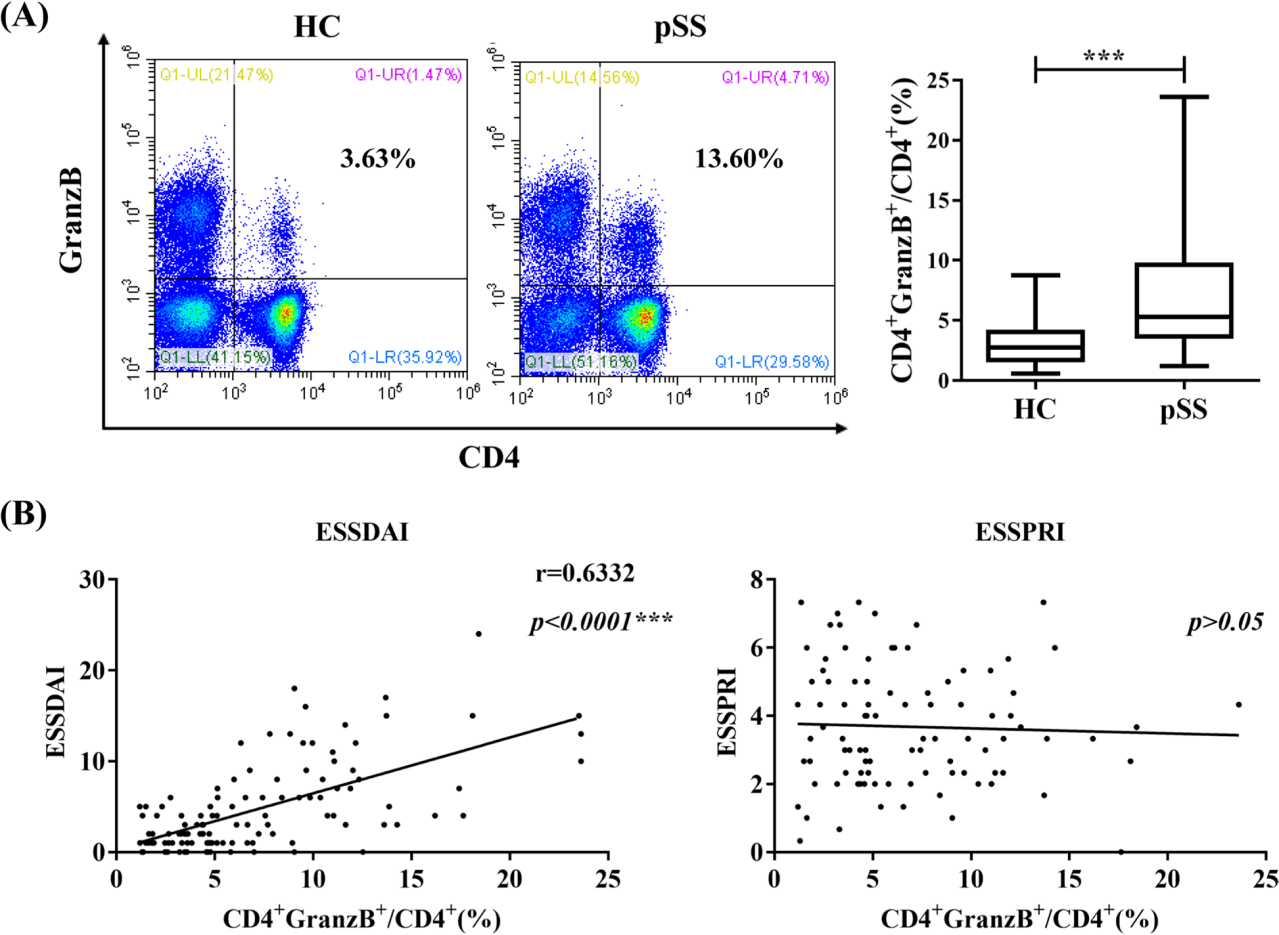
Elevated frequency of CD4+GranzB+CTLs and its correlation with ESSDAI and ESSPRI in pSS patients. **A** Percentage of CD4+GranzB+CTLs in pSS patients (*n* = 116) and HCs (*n* = 46). **B** Positive correlation of the percentage of CD4+GranzB+CTLs frequency with ESSDAI (*p* < 0.0001) and no correlation with ESSPRI in pSS patients (*p* > 0.05). CD4+GranzB+CTLs, circulating CD4+GranzB+cytotoxic T cells; ESSDAI, European League Against Rheumatism (EULAR) SS Disease Activity Index; ESSPRI, European League Against Rheumatism (EULAR) SS Patient Report Index; pSS, primary SS; HC, healthy controls. ***p < 0.001

One hundred and sixteen pSS patients and 46 healthy controls were recruited in this study. There were no significant differences in both mean age and sex distribution between pSS patients and healthy controls (Table 1). We examined peripheral CD4+GranzB+CTLs frequency in pSS patients and HCs. As shown in Fig. 1, flow cytometry analysis that the percentage of CD4+GranzB+CTLs were significantly upregulated in pSS patients than in healthy controls (7.1% ± 4.9% vs 3.1% ± 1.9%, *p* < 0.0001; Fig. 1A), and the CD4+GranzB+CTLs frequency was positively correlated with European League Against Rheumatism (EULAR) SS Disease Activity Index ( ESSDAI) (*r* = 0.6332, *p* < 0.0001), indicating that CD4+GranzB+CTLs is probably involved in the pathogenesis of pSS, while the CD4+GranzB+CTLs frequency had no correlation with European League Against Rheumatism SS Patient Report Index (ESSPRI) (*p* > 0.05).

### The percentage of CD4+GranzB+CTLs was significantly higher in pSS patients with extraglandular manifestations

**Fig. 2:**
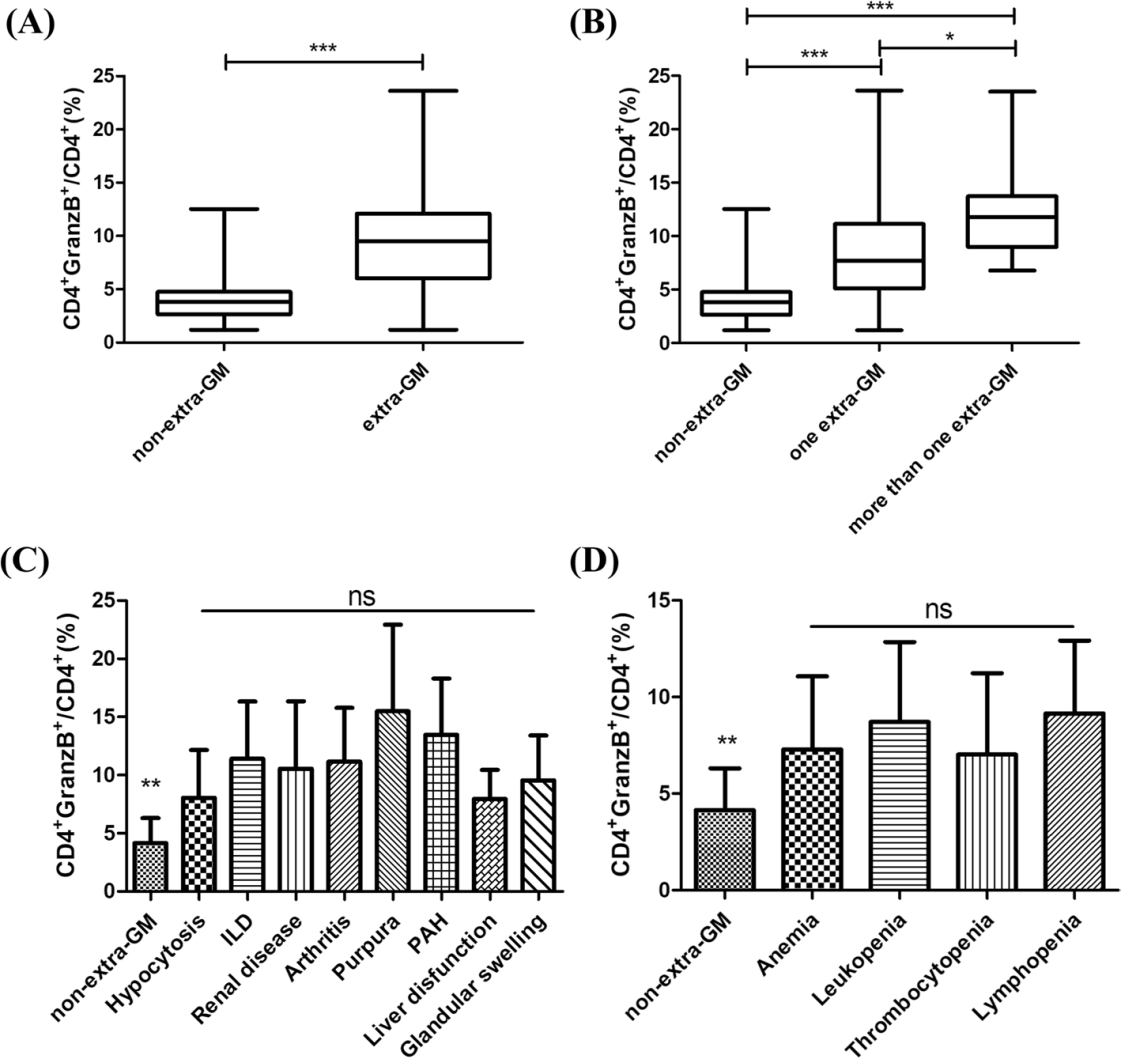
Higher percentage of CD4+GranzB+CTLs in pSS patients with extra-gland manifestations (extra-GM). **A** The frequency of CD4+GranzB+CTLs in pSS patients with non-extra-gland manifestations (non-extra-GM) (*n* = 55) and with extra-GM (*n* = 61). **B** The percentage of CD4+GranzB+CTLs in pSS patients with different number of extra-GM (non-extra-GM group *n* = 55, one extra-GM group *n* = 45, more than one extra-GM group *n* = 16). **C** Comparisons of the pSS patients with non-extra-GM, the percentage of CD4+GranzB+CTLs in pSS patients with different types of extra-GM (non-extra-GM group *n* = 55, hypocytosis group *n* = 29, ILD group *n* = 17, renal disease group *n* = 9, arthritis group *n* = 5, purpura group *n* = 5, PAH group *n* = 5, liver dysfunction group *n* = 3, glandular swelling group *n* = 3). **D** Comparisons of the pSS patients with non-extra-GM, the percentage of CD4+GranzB+CTLs in pSS patients with different types of various hypocytosis (non-extra-GM group *n* = 55; anemia group *n* = 7; leukopenia group *n* = 15; thrombocytopenia group *n* = 10; lymphopenia group *n* = 7). ***p < 0.001, **p < 0.01, *p < 0.05. ns, no significance

According to the pSS patients whether have systemic damages or not, we divided them into two groups: with or without extraglandular manifestations. The pSS patients only with sicca symptoms like dry eye or dry mouth were defined as the non-extraglandular manifestations group. Fifty-five cases among the 116 pSS patients were non-extraglandular manifestations, the mean percentage of CD4+GranzB+CTLs was 4.1% ± 2.2%.While the percentage was significantly elevated in the patients with various systemic damages (9.7% ± 5.3%, *p <* 0.0001, Fig. 2A). The more extraglandular manifestations existed, the higher percentage of CD4+GranzB+CTLs we got (Fig. 2B). We further had several subgroups in extraglandular manifestations group, then the frequency of CD4+GranzB+CTLs were analyzed. As shown in Fig. 2C and D, the percentage of CD4+GranzB+CTLs in pSS patients with different types of extraglandular manifestations were all markedly higher than those without extraglandular manifestations (*p* < 0.01). Respectively, the percentage of CD4+GranzB+CTLs in the pSS patients with arthritis (*n* = 5, 11.2% ± 4.6%), ILD (*n* = 17, 11.4% ± 4.9%), renal disease (*n* = 9, 10.5% ± 5.8%), purpura (*n* = 5, 15.5% ± 7.4%), PAH (*n* = 5, 13.5% ± 4.8%), autoimmune liver dysfunction (*n* = 3, 7.9% ± 2.5%), glandular swelling (*n* = 3, 9.5% ± 3.9%), hypocytosis (*n* = 29, 8.1% ± 4.1%), leukopenia (*n* = 15, 8.1% ± 4.1%), anemia (*n* = 7, 7.3% ± 3.8%), thrombocytopenia (*n* = 10, 7.0% ± 4.2%), and lymphopenia (*n* = 7, 9.1% ± 3.8%). But it was no significant difference among these subgroups with various extraglandular manifestations.

### Correlation of the percentage of CD4+GranzB+CTLs with other laboratory observations and Foci Index (FI)

**Fig. 3:**
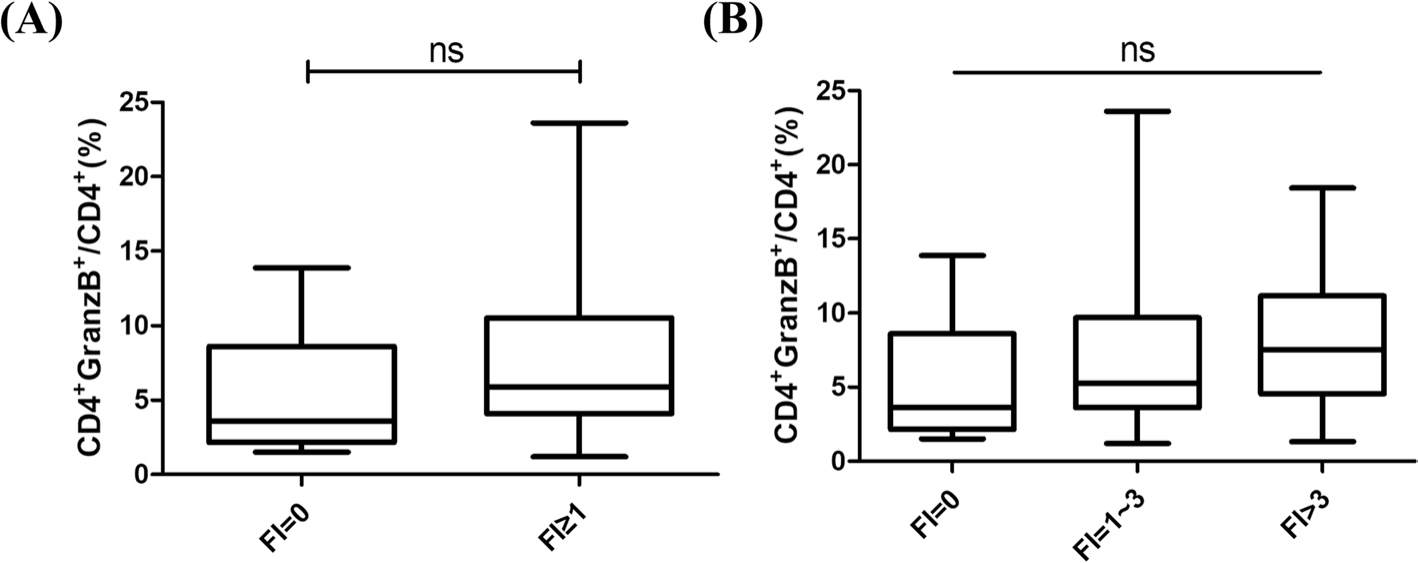
Comparison of the percentage of CD4+GranzB+CTLs among pSS patients with different Foci Index. **A** The pSS patients with lymphocytic infiltration had higher percentage of CD4+GranzB+CTLs in the peripheral blood (FI = 0, *n* =9; FI ≥ 1, *n* = 94). **B** The percentage of CD4+GranzB+CTLs were gradually elevated with the grade of Foci Index (FI = 0, *n* = 9; FI = 1~3, *n* = 70; FI > 3, *n* = 17). ns, no significance

To further determine the relationship between peripheral CD4+GranzB+CTLs frequency and laboratory test results including the titers of ANA, A-SSA, A-SSB, ESR, RF, Ig levels, complement, and different types of lymphocyte, we used correlation analysis and Student’s *t*-test to analyze quantitative and categorical data (Tables 2 and 3). It was found that no significant correlations were found between serum peripheral CD4+GranzB+CTLs and the laboratory values except ESR and CD8+GranzB+CTLs. The Foci Index (FI) was recorded as the number of foci per 4 mm^2^ of LSGs. We divided the pSS patients into 3 groups according to their FI. The percentage of CD4+GranzB+CTLs were gradually elevated with the grade of FI (FI (0), *n* = 9, 5.6% ± 4.0%; versus FI (1–3), *n* = 70, 7.1% ± 4.7%; versus FI (3), *n* = 17, 8.1% ± 4.6%). Although it was not found the significant difference among these three groups, we can find the rising trend which shows the elevated frequency of CD4+GranzB+CTLs were associated with increased severity of lymphocytic infiltration (Fig. 3).

**Table 2:** Correlation of the percentage of CD4+GranzB+CTLs between pSS patients with laboratory values (quantitative data)

	*r*	*p*
IgG	0.1161	0.2185
IgA	0.0146	0.8790
IgM	0.1504	0.1150
C3	0.0385	0.7159
C4	0.0258	0.8113
**ESR**	**0.2056**	0.0456*
CD4+ T cell%	0.0940	0.4603
CD8+ T cell%	0.1896	0.1336
NK cell%	0.0776	0.5084
CD19^+^ B cell%	0.0017	0.9881
**CD8+ GranzB+CTLs%**	**0.5491**	< 0.0001***

*p < 0.05, ***p < 0.001

**Table 3:** Comparison of the percentage of CD4+GranzB+CTLs between pSS patients with laboratory values (categorical data)

Parameter	Normal (mean ± SD)	Abnormal (mean ± SD)	*p* value
ANA	7.3 ± 3.0	7.1 ± 5.0	0.940
A-SSA	6.3 ± 5.3	7.3 ± 4.9	0.442
A-SSB	7.0 ± 4.7	7.4 ± 5.4	0.712
RF	7.0 ± 4.6	8.3 ± 5.7	0.198

### The percentage of CD4+GranzB+CTLs was independently related to ESSDIA and extraglandular manifestations in pSS patients

In the multivariate regression analysis, we take CD4+GranzB+CTLs as the dependent variable, while age, gender, and pSS-relevant factors such as IgG, IgA, IgM, C3, C4, ESR, anti-SSA, anti-SSB, CD8+GranzB+CTLs, ESSPRI, ESSDAI, PGA, and extraglandular manifestations were as independent variables. After excluding the other risk factors, CD4+GranzB+CTLs were still related to CD8+GranzB+CTLs, ESSDIA, and extraglandular manifestations independently (*p* < 0.05) (Table 4).

**Table 4:** Multivariate analysis of CD4+GranzB+CTLs influenced by pSS-relevant factors

	Regression coefficient	Standard error	*t*-statistics	*p* value	95% CI
CD8+GranzB+CTLs%	0.144	0.033	4.334	6.9E−5	0.077, 0.211
ESSDAI	0.256	0.122	2.095	0.041	0.011, 0.502
Extraglandular manifestations	2.612	1.268	2.059	0.045	0.065, 5.158

To understand GranzB+CTLs in pSS, we also analyzed the correlation between CD8+GranzB+CTLs and pSS-relevant factors. The similar results with CD4+GranzB+CTLs seemed to be found by the univariate analysis. The percentage of CD8+GranzB+CTLs also elevated in pSS patients than in HCs (37.4% ± 15.9% vs 51.3% ± 14.3%, *p* < 0.0001) and significantly related to ESSDAI (*r* = 0.3665, *p* < 0.0001) (showed in Sup Fig. 1) and higher in extraglandular manifestations group (*p* < 0.05). Further analysis of different types of extraglandular manifestations in pSS patients showed that the percentage of CD8+GranzB+CTLs were significantly elevated in the pSS patients with ILD, purpura, PAH, and autoimmune liver dysfunction (*p* < 0.05) (showed in Sup Fig. 2) and correlated with IgG, ESR and NK cell% (*p* < 0.05) (showed in Sup Table 1&2). However, by multivariate regression analysis, it manifested that CD8+GranzB+CTLs were just independently correlated with CD4+GranzB+CTLs and ESR (*p* < 0.05), instead of ESSDAI or extraglandular manifestations (Table 5).

**Table 5:** Multivariate analysis of CD8+GranzB+CTLs influenced by pSS-relevant factors

	Regression coefficient	Standard error	*t*-statistics	*p* value	95% CI
CD4+GranzB+CTLs%	1.784	0.313	5.699	5.7E−7	1.156, 2.412
ESR	0.172	0.075	2.304	0.025	0.022, 0.322

### The percentage of CD4+GranzB+CTLs was an independent risk factor for extraglandular manifestations

We used logistic regression analysis to further explain whether CD4+GranzB+CTLs could be in predictive value for extraglandular involvements to pSS patients compared with other traditional factors. Extraglandular involvements in pSS patients were the dependent variable, while age, gender, and pSS-related factor such as IgG, IgA, IgM, C3, C4, ESR, anti-SSA, anti-SSB, ESSPRI, ESSDAI, PGA, CD8+GranzB+CTLs, and CD4+GranzB+CTLs were as independent variables. As shown in Table 6, CD4+GranzB+CTLs and ESSDAI were the independent risk factors for extraglandular involvements in pSS patients (OR were 1.928 and 5.217, respectively) (*p* < 0.05).

**Table 6:** Logistic regression analysis of extraglandular manifestations

	Partial regression coefficent	Standard error	*p* value	OR	OR 95% CI
Female	− 6.071	2.82	0.031	0.002	0.000, 0.581
ESSDAI	1.652	0.64	0.01	5.217	1.489, 18.278
CD4+GranzB+CTLs%	0.656	0.3	0.029	1.928	1.071, 3.470

### High expression of CD4+GranzB+CTLs in the labial salivary glands (LSGs) from the pSS patients

**Fig. 4:**
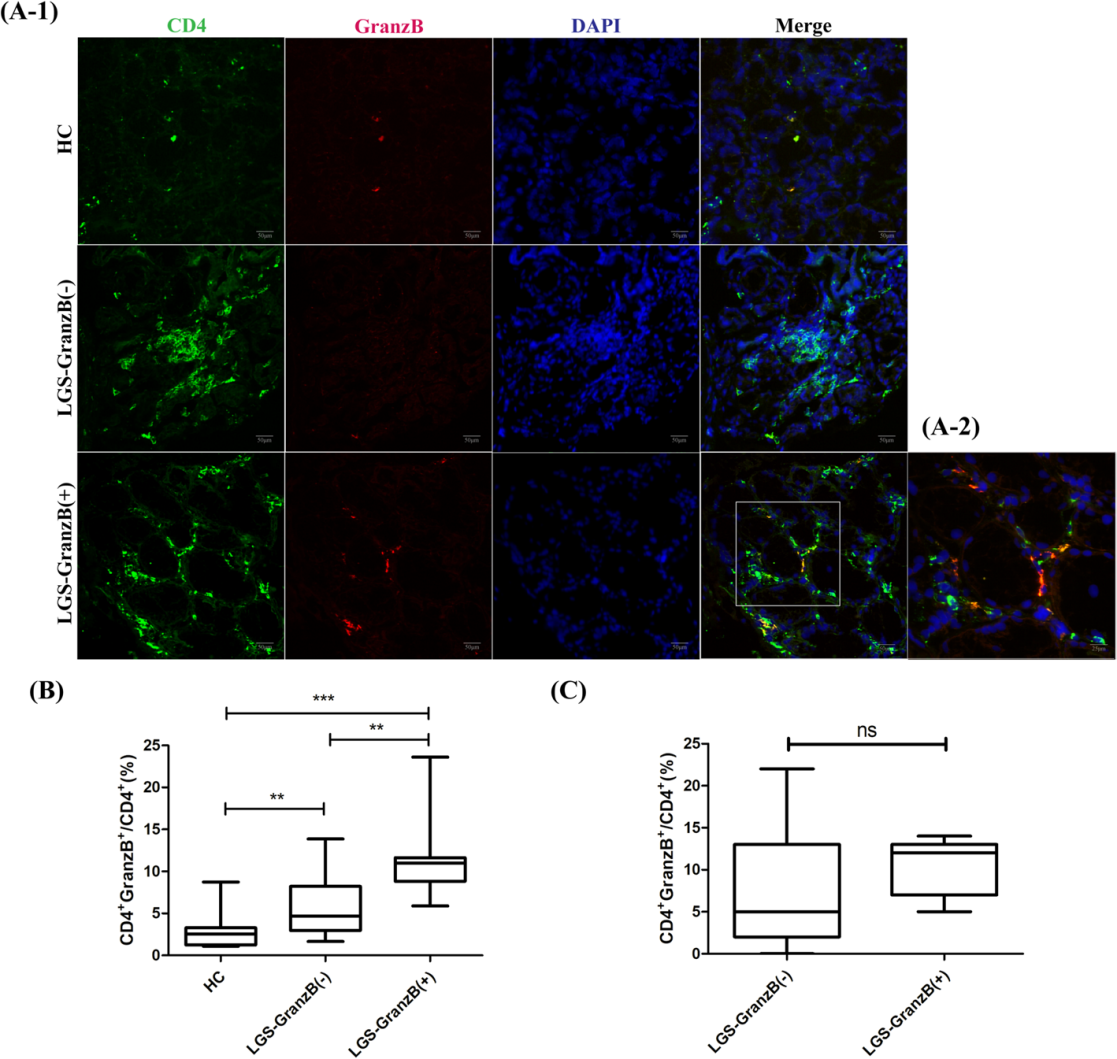
Expression of CD4+GranzB+CTLs in the labial salivary glands (LSGs). **A-1** Immunofluorescence staining of CD4 (green), GranzB (red), and DAPI (blue) in LSGs for a healthy control (HC), a pSS patient with non-expression of CD4+GranzB+CTLs (LGS-GranzB(−)), and a pSS patient with high expression of CD4+GranzB+CTLs (LGS-GranzB(+)). Scale bars, 50μm. **A-2** The image in the white box of **A-1** at high power. Scale bars, 20 μm. **B** Comparison of CD4+GranzB+CTLs% in the peripheral blood in HC (*n* = 13), LGS-GranzB(−) (*n* = 22), and LGS-GranzB(+) groups (*n* = 7). Error bars represent SD. *p* values are based on Student’s *t*-test. **C** Comparison of ESSDAI in the LGS-GranzB(−) (*n* = 22) and LGS-GranzB(+) groups (*n* = 7). Error bars represent SD. *p* values are based on Student’s *t*-test. ***p < 0.001, **p < 0.01. ns, no significance

To evaluate the local effect of CD4+GranzB+CTLs in LSGs and the correlation with the peripheral CD4+GranzB+CTLs in the pSS patients, we applied histologic and immunofluorescence analyses to determine the expression of CD4+GranzB+CTLs in LSGs. The freshly explanted lower lip biopsy specimens were sectioned and stained with anti-CD4 and anti-GranzB antibodies in 29 cases of the 116 pSS patients and 13 healthy control, then immunofluorescence analysis. Seven specimens from pSS patients exhibited distinct expression of CD4+GranzB+CTLs, while none of the LSGs from the healthy controls exhibited CD4+GranzB+CTLs expression. Compared to non-expression of CD4+GranzB+CTLs in LSGs (LGS-GranzB(−)), the pSS patients with high expression of CD4+GranzB+CTLs in LSGs (LGS-GranzB(+)) had significantly higher percentage CD4+GranzB+CTLs in peripheral blood (HC, *n* = 13, 2.7% ± 2.1%, versus LGS-GranzB(−), *n* = 22, 5.9% ± 3.8%, versus LGS-GranzB(+), *n* = 7, 11.7% ± 5.6%) (Fig. 4). We defined ESSDAI score ≥ 5 as disease activity. Chi-square test was performed on the two groups (LGS-GranzB(−) and LGS-GranzB(+)), and it was found that the proportion of pSS patients with disease activity was 100% in LGS-GranzB(+) group, which was much higher than it in the LGS-GranzB(−) group (45.45%) (*p* < 0.05) (Table 7).

**Table 7:** Disease activity between the LGS-GranzB(−) and LGS-GranzB(+) groups

	ESSDAI < 5	ESSDAI ≥ 5	Total
LGS-GranzB(−), *n* (%)	10 (45.45%)	12 (54.54%)	22
LGS-GranzB(+), *n* (%)	0 (0%)	7 (100%)	7

### High expression of CD4+GranzB+CTLs in the pSS patients can evaluate ESSDAI and extraglandular manifestations by receiver operating characteristic (ROC) analysis

**Fig. 5:**
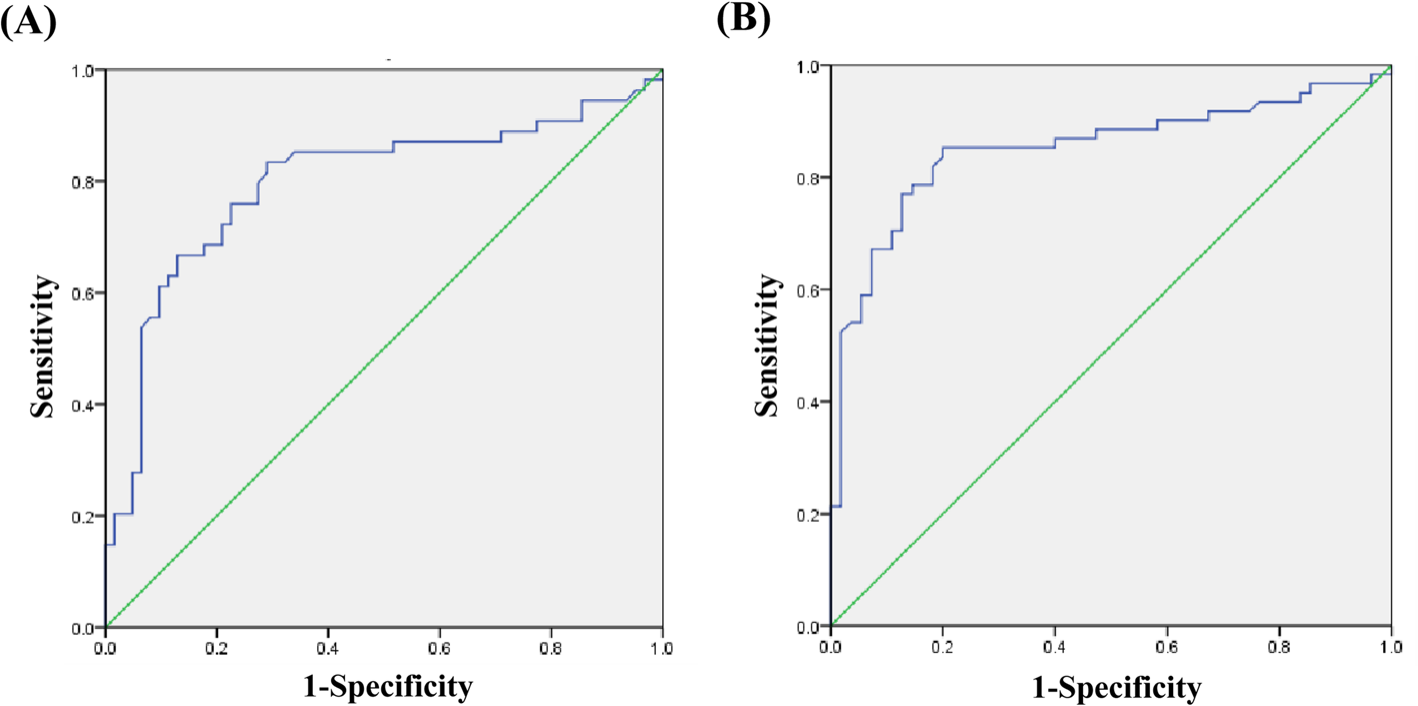
Receiver operating characteristic (ROC) curve of the frequency of CD4+GranzB+CTLs to predict ESSDAI and extraglandular manifestations response

To obtain the best predictive efficiency between the high expression of CD4+GranzB+CTLs and the disease activity and severity in the pSS patients, the receiver operating characteristic (ROC) curve was used to evaluate the predictive value of the frequency of CD4+GranzB+CTLs in predicting response to ESSDAI and extraglandular manifestations. ESSDAI ≥ 5 was defined as active phase of disease. Figure 5A shows the ROC curve for CD4+GranzB+CTLs predicting ESSDAI ≥ 5. The area under the curve (AUC) was 0.796 (95% CIs, 0.709–0.884) and CD4+GranzB+CTLs% = 4.865% was the best diagnostic threshold for the ROC curve. The corresponding sensitivity and specificity were 83.3% and 71%, respectively. Interestingly, also with a cutoff value of 4.865, the frequency of CD4+GranzB+CTLs could predict the patients with pSS in response to extraglandular manifestations with a sensitivity of 85.2% and a specificity of 80% (AUC = 0.851; 95% CIs, 0.777–0.925) (Fig. 5B).

## Discussion

pSS is a highly heterogeneous disease which not only damages exocrine gland but also injures other vital organs, like pulmonary, neurological, and renal, leading to death. This heterogeneity is also reflected in the glandular autoimmune lesions. The main components of the ectopic germinal center formed in the glandular lesions of pSS are T cells, which are considered to play a vital role in the pathogenesis of pSS [19].

CD4 CTLs can develop from Th0, Th1, Th2, Th17, and Treg effector subsets apparently. It is generally known that the master regulator of Th1 differentiation is the transcription factor T-bet which could induce IFNγ production. T-bet also produces the expression of perforin and granzyme B; both of them are required for CD8 CTLs cytotoxicity [9]. This indicates that CD4 CTLs utilize the transcriptional program of the CD8 lineage while retaining CD4 expression and exhibited substantial heterogeneity in their degree of cytotoxicity [20].

Croia et al. [21] found that chronic EBV infection which is selectively correlated with ectopic lymphoid structures in the salivary glands of patients with pSS seems to contribute to local growth and differentiation of disease-specific autoreactive B cells. Association of lytic EBV reactivation, the prevalent granzyme B-positive population within B cell follicles coexpressed CD4 or CD8 in ectopic lymphoid structures in pSS salivary gland tissue, suggesting that a subset of CD4+GranzB+CTLs involved in the process of the ectopic lymphoid structures in pSS.

However, the mechanism of CD4+GranzB+CTLs activation has not been the first study to reveal a close correlation between the frequency CD4+GranzB+CTLs and the disease activity in patients with pSS. Our data have clearly shown that the percentage of CD4+GranzB+CTLs were significantly upregulated in pSS patients than in healthy controls and was positively correlated with ESSDAI and ESR. In addition, the percentage of CD4+GranzB+CTLs was markedly higher in pSS patients with extraglandular manifestations including secondary interstitial lung disease, renal damage, arthritis, and autoimmune liver dysfunction, as well as glandular swelling. In multivariate analysis, we found that CD4+GranzB+CTLs are independently related to ESSDAI and extraglandular manifestations in pSS patients. We further analyzed the related factors in pSS patients with extraglandular manifestations, and after logistic regression analysis, ESSDAI and CD4+GranzB+CTLs were founded to be the independent risk factors for organ lesions (OR values were 5.217 and 1.928, respectively).

To further understand the role of GranzB+CTLs in pSS, we also analyzed the frequency of CD8+GranzB+CTLs and the relationship between CD8+GranzB+CTLs and CD4+GranzB+CTLs in pSS patients. CD8+GranzB+CTLs in pSS patients seemed to show similar effects as CD4+GranzB+CTLs by using mono-factor analysis, while, after excluding the classical risk factors in pSS patients by using multivariate regression analysis, CD8+GranzB+CTLs only showed correlation with CD4+GranzB+CTLs and ESR independently, having no association with ESSDAI and extraglandular manifestations in pSS patients. These results may indicate that CD8+GranzB+CTLs were not an independent risk factor for organ lesions in pSS patients. Our findings further illustrated that the percentage of CD4+GranzB+CTLs in peripheral blood was a better indicator than CD8+GranzB+CTLs for the assessment of disease activity in pSS patients. Further studies are warranted to determine whether CD4+GranzB+CTLs may play an even more important role than CD8+GranzB+CTLs in the pathogenesis of pSS.

Here, we provide new evidence on the involvement of CD4+GranzB+CTLs in autoimmune progression in pSS patients and their implication in predicting extraglandular manifestations of pSS. However, we also found that female was an independent protective factor for organ lesion in pSS (OR = 0.002), which may be partially attributed to the small number of male patients enrolled in this study (*n* = 6) and their disease activity.

Besides tumors and chronic infections, CD4+GranzB+CTLs were also found in some autoimmune or chronic inflammatory diseases, including RA [22], atherosclerosis [23], Graves’ disease [24], sporadic inclusion body myositis [25], and Wegener’s granulomatosis [26]. Liesbet et al. [27] discovered that CD4+GranzB+CTLs were capable of migrating to sites of inflammation and lead to local tissue damage. While protecting against tumors and chronic infections, CD4+GranzB+CTLs also involve in the progression of autoimmunity disease. Their studies provided the direct link between the presence of CD4+GranzB+CTLs and multiple sclerosis (MS) disease severity. CD4+GranzB+CTLs could drive the progression of MS and were correlated with worse clinical outcome and prognosis in MS patients, while inhibiting the production of CD4+GranzB+CTLs could slow down the progression of MS disease. Furthermore, in an animal model for multiple sclerosis, they also showed that CD4+GranzB+CTLs are correlated with inflammation, demyelination, and disability.

In our study, CD4+GranzB+CTLs could be observed in the lymphocytic foci and periductal areas of the LSGs. On the contrary, the LSGs of healthy control subjects did not express CD4+GranzB+CTLs. We also analyzed the relations and degrees between labial salivary glands which infiltrated CD4+GranzB+CTLs in 29 pSS patients and their ESSDAI and found that the ESSDAI score in LSG-GranzB(+) group was higher than in LSG-GranzB(−) group. One hundred percent of pSS patients in LSG-GranzB(+) group had active disease (ESSDAI ≥ 5), while only 45.45% in LSG-GranzB(−) group. Although the difference between these two groups was not significant, it also showed the trend and might be caused by the limited numbers of LSG-GranzB(+) group (*n* = 7). It is possible that cytotoxicity by CD4+CTL becomes more effective when CD8+CTL activity is impaired in association with cytokines and chemokines might make the discrepancy between peripheral blood mononuclear cells and gland tissues. Further studies will be needed to conform. In addition, ESSDAI score was mainly related to extraglandular manifestations in pSS patients, which further suggested that local infiltration of CD4+GranzB+CTLs might be involved in pSS target organ damage, leading to disease activity. We also found that there was a correlation between the infiltration of CD4+GranzB+CTLs in the labial salivary gland and in peripheral blood, suggesting that the percentage of CD4+GranzB+CTLs in peripheral blood may represent the infiltration of them in local tissues. This was also consistent with the notion that CD4+GranzB+CTLs were correlated with extraglandular manifestations of pSS in our previous analysis.

A lot of evidence which was found in animal models and human studies indicate that chronic dry eye disease is often correlated with T cell infiltration of the conjunctiva [28]. T cell infiltration can also been detected in dry eye patients with or without a systemic autoimmune disease, and an adaptive CD4+ T cell autoimmune response was following the initial innate immune response to dryness in mice exposed to desiccating stress and patients with pSS and non-SS-associated aqueous tear deficiency [29, 30].

Further, the identification of the reason for the epithelial activation associated with pSS and the elucidation of interactions between epithelial and immune cell are extremely important for the understanding of pSS pathogenesis and the development of effective therapeutic interventions.

At the meantime, our study established receiver operating characteristic (ROC) analysis for the first time to evaluate the frequency of CD4+GranzB+CTLs as the prognostic parameters in differentiating whether pSS patients have extraglandular manifestations or not. The receiver operating characteristic (ROC) analysis showed that the high frequency of CD4+GranzB+CTLs could better indicate the pSS patients have extraglandular manifestations and multisystem damage. Conducting a ROC analysis for patients with pSS patients suspected of having systemic damages presents early stage prognostic value compared with traditional ESSDAI. It also helps clinicians make different treatment plans.

## Conclusions

Our investigations have revealed a positive correlation of CD4+GranzB+CTLs with the disease activity and severity in pSS patients. We provide new evidence indicating CD4+GranzB+CTLs, in addition to CD8+GranzB+CTLs, may be involved in the pathophysiology of pSS, which may serve as a new biomarker to evaluate the disease activity and severity of pSS.

## Supplementary Information

**Additional file 1: Sup Fig. 1.** Elevated frequency of CD8+GranzB+CTLs and its correlation with ESSDAI and ESSPRI in pSS patients: (A) Percentage of CD8+GranzB+CTLs in pSS patients (n = 116) and HCs (n = 46). (B) Positive Correlation of the percentage of CD8+GranzB+CTLs frequency with ESSDAI and no correlation with ESSPRI in pSS patients. CD8+GranzB+CTLs: circulating CD8+GranzB+cytotoxic T cells; ESSDAI: European League Against Rheumatism (EULAR) SS Disease Activity Index; pSS: primary SS; HC: healthy controls. *** *p* < 0.001. **Sup Fig. 2.** Higher percentage of CD8+GranzB+CTLs in pSS patients with extra-glandular manifestations (extra-GM): (A) The frequency of CD8+GranzB+CTLs in pSS patients with non-extra-GM (n = 55) and with extra-GM (n = 61);(B) The percentage of CD8+GranzB+CTLs in pSS patients with different number of extra-GM (non-extra-GM group n=55; one extra-GM group n=45; more than one extra-GM group n=16); (C) Comparisons of the pSS patients with non-extra-GM, the percentage of CD8+GranzB+CTLs in pSS patients with different types of extra-GM (non-extra-GM group n=55; ILD group n=17; Purpura group n=5; PAH group n=5; Liver disfunction group n=3;Renal disease group n=9; Arthritis group n=5; Glandular swelling group n=3; Hypocutosis group n=29). *** *p* < 0.001, ** *p* < 0.01, * *p* < 0.05, ns: no significance. **Sup Table 1.** Correlation of the percentage of CD8+ GranzB+CTLs between pSS patients with laboratory values (quantitative data). **Sup Table 2.** Comparison of the percentage of CD8+ GranzB+CTLs between pSS patients with laboratory values (categorical data).

## Data Availability

All data generated or analyzed during this study are included in this published article.
