# Supplementary material for: Correlation of peripheral CD4+GranzB+CTLs with disease severity in patients with primary Sjögren’s syndrome
Source: Arthritis Res Ther. 2021 Oct 12;23:257. doi: 10.1186/s13075-021-02632-6 (PMC8507116; doi:10.1186/s13075-021-02632-6)
Supplement: Supplementary file 1 — Additional file 1: Sup Fig. 1. Elevated frequency of CD8+GranzB+CTLs and its correlation with ESSDAI and ESSPRI in pSS patients: (A) Percentage of CD8+GranzB+CTLs in pSS patients (n = 116) and HCs (n = 46). (B) Positive Correlation of the percentage of CD8+GranzB+CTLs frequency with ESSDAI and no correlation with ESSPRI in pSS patients. CD8+GranzB+CTLs: circulating CD8+GranzB+cytotoxic T cells; ESSDAI: European League Against Rheumatism (EULAR) SS Disease Activity Index; pSS: primary SS; HC: healthy controls. *** p < 0.001. Sup Fig. 2. Higher percentage of CD8+GranzB+CTLs in pSS patients with extra-glandular manifestations (extra-GM): (A) The frequency of CD8+GranzB+CTLs in pSS patients with non-extra-GM (n = 55) and with extra-GM (n = 61);(B) The percentage of CD8+GranzB+CTLs in pSS patients with different number of extra-GM (non-extra-GM group n=55; one extra-GM group n=45; more than one extra-GM group n=16); (C) Comparisons of the pSS patients with non-extra-GM, the percentage of CD8+GranzB+CTLs in pSS patients with different types of extra-GM (non-extra-GM group n=55; ILD group n=17; Purpura group n=5; PAH group n=5; Liver disfunction group n=3;Renal disease group n=9; Arthritis group n=5; Glandular swelling group n=3; Hypocutosis group n=29). *** p < 0.001, ** p < 0.01, * p < 0.05, ns: no significance. Sup Table 1. Correlation of the percentage of CD8+ GranzB+CTLs between pSS patients with laboratory values (quantitative data). Sup Table 2. Comparison of the percentage of CD8+ GranzB+CTLs between pSS patients with laboratory values (categorical data). [file 13075_2021_2632_MOESM1_ESM.pdf]

Supplemental Information

Results of univariate analysis of CD8+GranzB+CTLs and pSS relevant factors were showed below:

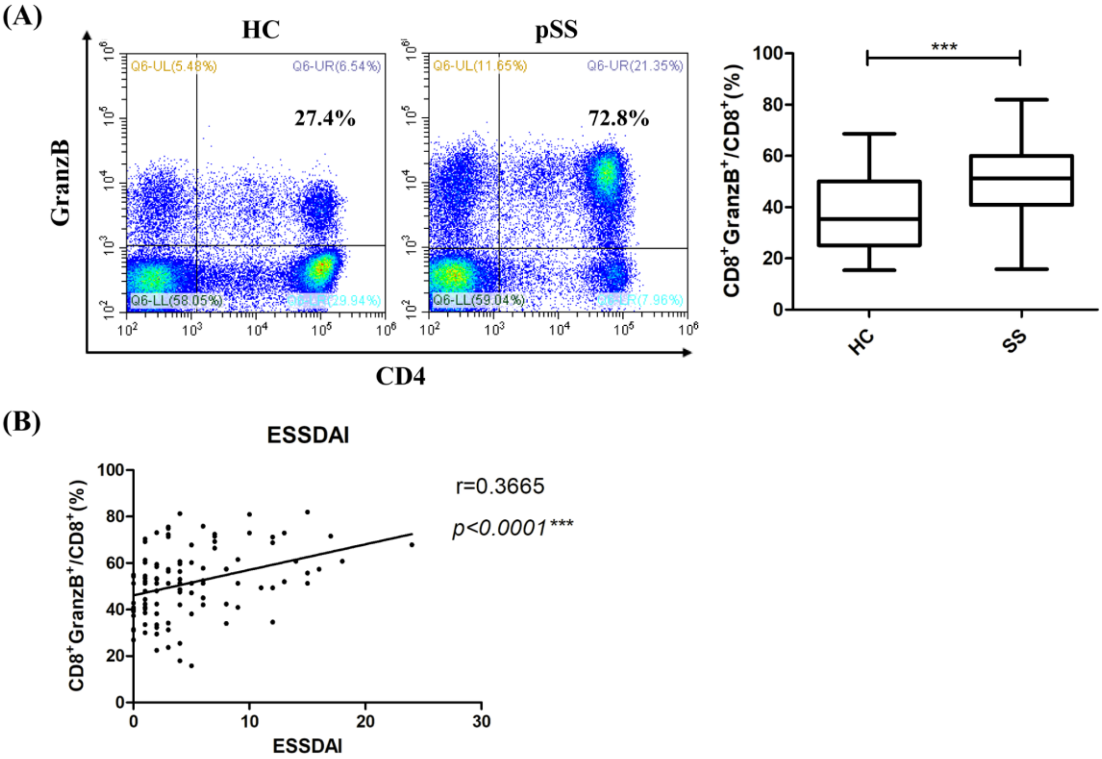

Sup Fig. 1 Elevated frequency of CD8+GranzB+CTLs and its correlation with ESSDAI and ESSPRI in pSS patients: (A) Percentage of CD8+GranzB+CTLs in pSS patients (n = 116) and HCs (n = 46). (B) Positive Correlation of the percentage of CD8+GranzB+CTLs frequency with ESSDAI and no correlation with ESSPRI in pSS patients. CD8+GranzB+CTLs: circulating CD8+GranzB+cytotoxic T cells; ESSDAI: European League Against Rheumatism (EULAR) SS Disease Activity Index; pSS: primary SS; HC: healthy controls. *** p<0.001

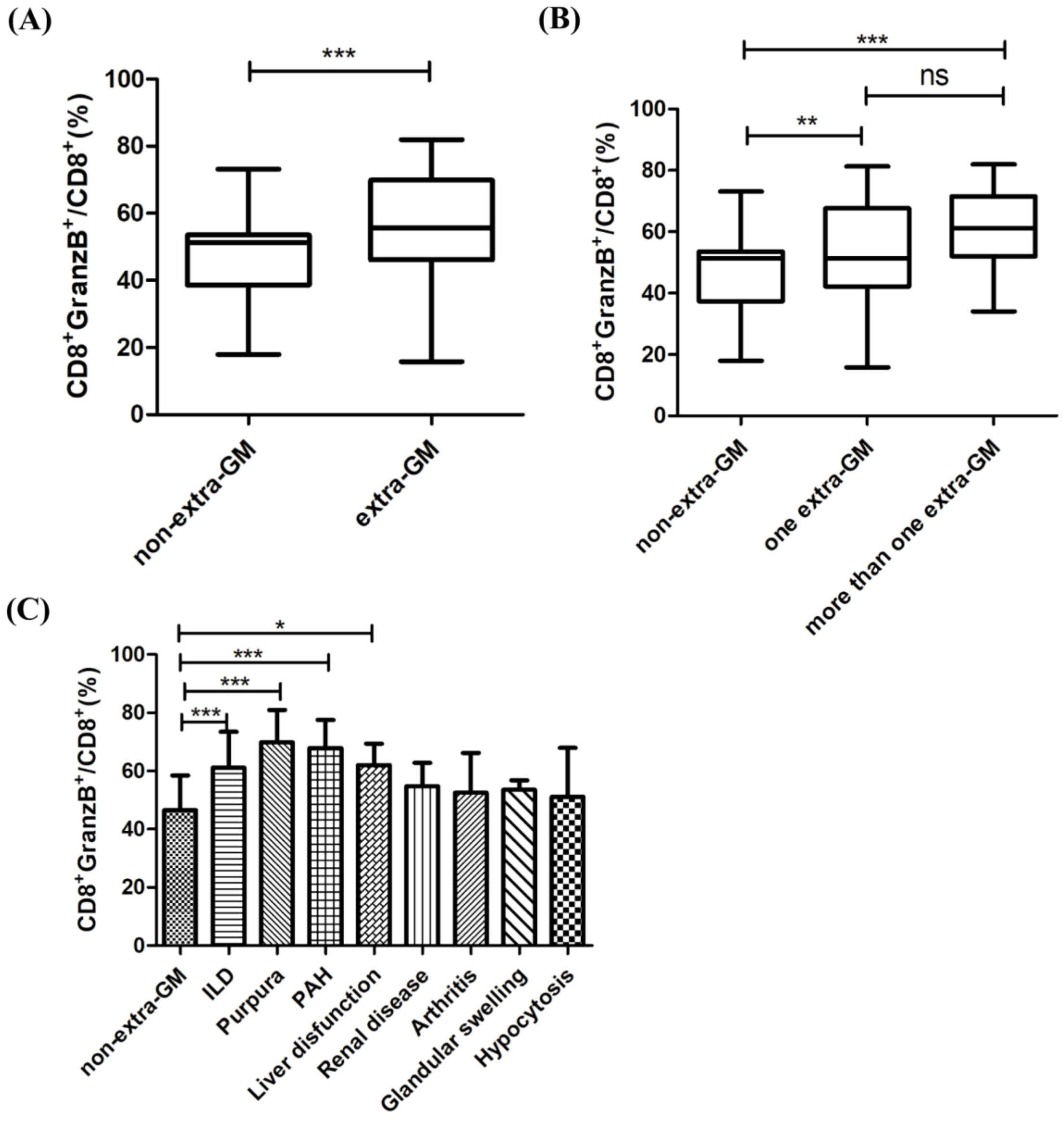

Sup Fig. 2 Higher percentage of CD8+GranzB+CTLs in pSS patients with extra-glandular manifestations(extra-GM): (A)The frequency of CD8+GranzB+CTLs in pSS patients with non-extra-GM(n = 55) and with extra-GM (n = 61);(B)The percentage of CD8+GranzB+CTLs in pSS patients with different number of extra-GM(non-extra-GM group n=55; one extra-GM group n=45; more than one extra-GM group n=16); (C)Comparisons of the pSS patients with non-extra-GM, the percentage of CD8+GranzB+CTLs in pSS patients with different types of extra-GM(non-extra-GM group n=55; ILD group n=17; Purpura group n=5; PAH group n=5; Liver disfunction group n=3;Renal disease group n=9; Arthritis group n=5; Glandular swelling group n=3; Hypocutosis group n=29). *** p<0.001, ** p<0.01, * p<0.05, ns: no significance.

Sup Table 1 Correlation of the percentage of CD8+ GranzB+CTLs between pSS patients with laboratory values(quantitative data)

	r	*p*
**IgG**	**0.1944**	**0.0382***
IgA	0.0478	0.6185
IgM	0.0575	0.5429
C3	0.1446	0.1690
C4	0.0390	0.7117
**ESR**	**0.2505**	**0.0144***
$CD4^+$T cell%	0.0020	0.9873
$CD8^+$Tcell%	0.1822	0.1493
**NK cell%**	**0.2297**	**0.0474***
$CD19^+$ B cell%	0.0209	0.8587

Sup Table 2 Comparison of the percentage of CD8+ GranzB+CTLs between pSS patients with laboratory values(categorical data)

Parameter	CD8+ GranzB+CTLs(%)		*p* value
	Normal(Mean ± SD)	abnormal (Mean ± SD)	
ANA	51.1±6.1	51.3±14.5	0.980
A-SSA	49.7±13.5	51.6±14.5	0.608
A-SSB	49.4±14.6	54.6±13.4	0.060
RF	51.4±15.3	53.4±15.1	0.546
